# Genetic and environmental risk factors of Parkinsonism

**DOI:** 10.1007/s00702-025-03058-z

**Published:** 2026-04-06

**Authors:** Petr Kanovsky, Katerina Mensikova, Pavel Cupr, Radek Vodicka, Kristyna Kolarikova, Tereza Strnadova, Sarah Elizabeth Victoria Cook, Dorota Sebelova-Konickova, Jana Klanova, Carlo Colosimo, Raymond Rosales

**Affiliations:** 1 Department of Neurology, University Hospital Olomouc, Olomouc, Czech Republic; 2 Department of Neurology, Faculty of Medicine and Dentistry, Palacky University, Olomouc, Czech Republic; 3 RECETOX, Faculty of Science, Masaryk University, Brno, Czech Republic; 4 Department of Clinical Genetics, University Hospital Olomouc, Olomouc, Czech Republic; 5 Department of Neurology, University Hospital Santa Maria, Terni, Italy; 6 Department of Neurology and Psychiatry, University of Santo Tomás, Manila, Philippines

**Keywords:** Parkinson´ disease - alpha-synuclein - genetics - environment - risk factors

## Abstract

Parkinsonian disorders comprise a broad spectrum of neurodegenerative diseases with a wide variety of pathogenetic processes. These processes lead to the formation of pathological proteins, resulting in the brain diseases called synucleinopathies, tauopathies or TDP-43 proteinopathies. There is currently growing support for the hypothesis that genetic variants explain a significant fraction of the etiology of apparently sporadic parkinsonian disorders. Genetic risk factors can be stratified according to the metabolic or structural processes that can lead to cellular disturbance; these processes involve protein aggregation, protein and membrane trafficking, stabilization of the neurite structure, prion-like transmission of pathological proteins, ubiquitin–proteasome system balance, mitophagy, lysosome autophagy, synaptic functions, and dopamine transmission. Regarding the environmental risk factors, there are several substances that have been supposed of being a risk for the development of neurodegenerative proteinopathy and Parkinsonism, mainly the agents used in agriculture and the textile industry. The most important and most frequently studied are pesticides and trichlorethylene. Beside the globally ubiquitous substances which are supposedly neurotoxic and exposure to which can cause manifestations of Parkinsonism, there are more geographically (regionally) specific substances, which cause (or quite recently caused) the manifestation of endemically present Parkinsonism. Among ten types of endemic Parkinsonism, three of them are thought to have an environmental cause: Western Pacific Parkinsonism, Caribbean Parkinsonism, and North France cluster of atypical Parkinsonism.

## Introduction

Parkinsonian disorders is an umbrella term which covers a heterogenous group of diseases. Their common denominator is the presence of parkinsonian syndrome as a dominant feature of clinical presentation. The disease cause in almost all parkinsonian disorders is neurodegeneration, however the pathological process leading to neurodegenerative changes of brain tissue might be different.

Nevertheless, the final process (apoptosis, autophagy or necrosis) led to the formation of pathological proteins. Thus, the diseases are then labelled as neurodegenerative proteinopathies and nosologically classified on the quality and distribution of protein: neuronal cytoplasm, neuronal nucleus, glial cytoplasm or extracellular. Neurodegenerative proteinopathies are diagnosed using clinical, genetic and pathological approaches. Due to the rare existence of “definite” clinico–genomic correlation, definite diagnosis is confirmed at autopsy in most of these disorders. The current classification of neurodegenerative proteinopathies (Forrest and Kovacs 2025) is in Table 1.

**Table 1:** Classification of neurodegenerative diseases (Forrest and Kovacs 2025)

Proteinopathy	Protein	Disease/subtype	Pathological hallmark/structure
α-synuclein proteinopathy	α-synuclein	Lewy body disease/Parkinson´s disease	Lewy pathology, Lewy bodies
		Lewy body disease/Dementia with Lewy bodies	Lewy pathology, Lewy bodies
		Multiple system atrophy (cerebellar type)	MSA pathology
		Multiple system atrophy (Parkinsonian type)	MSA pathology
Tau and Aß proteinopathy (Alzheimer´s disease related)	Tau (3R and 4R)	Alzheimer’s disease	3R and 4R tauopathy, ß-amyloid deposits, senile plaques
	Aß	Alzheimer’s disease	Amyloid-ß_42_ filaments in brain cortex Amyloid-ß_40_ aggregates in meninges
Tau proteinopathy	Tau (3R)	Pick’s disease	Pick´s pathology, Pick bodies
	Tau (4R)	Corticobasal degeneration	CBD pathology
		Argyrophylic grain disease	AGD pathology
		Progressive supranuclear palsy	PSP pathology
	Tau (3R and 4R)	Globular glial tauopathy	GGT pathology
		Primary age-related tauopathy	Alzheimer’s disease tau pathology
TDP-43 proteinopathy	TDP-43	FTLD-TDP; type A-D (bvFTD)	Filaments from two cases with FTLD-TDP type A and type C pathology
		Motor neuron disease (amyotrophic lateral sclerosis)	Filaments from two cases with FTLD-MND pathology
		TDP-43 in Alzheimeir’s disease LATE-NC stages 1–3	??

### α-synucleinopathies

Nosological term for the group of neurodegenerative diseases characterised by the presence of pathologically conformed oligomeric α-synuclein; this is present in the neural tissue as well as in other tissues, i.e. skin, gastrointestinal tract and endocrine tissues.

In the nervous system, it is present both intraneuronally and extraneuronally. Due to this fact, the α-synucleinopathies are categorised as intraneuronal (LBD–PD, DLB) and extraneuronal (MSA). α-synuclein forms Lewy bodies (LB) in LBD, and oligodendroglial cytoplasmic inclusions (GCI) in MSA where α-synuclein may also be present freely in the glial cytoplasm (Koga at el. 2021).

### Tauopathies

Roofing term for the group of neurodegenerative diseases which are characterised by the presence of pathologically conformed isoforms of tau protein; this is present only in the brain tissue (Kovacs et al. 2022). Tauopathies are taxonomically categorised into 3 groups: 3R-tauopathies (Pick´s disease or frontotemporal lobar degeneration, FTLD), 4R-tauopathies (progressive supranuclear palsy, PSP; corticobasal degeneration, CBD, argyrophilic grain disease, ARTAG), and 3 + 4R tauopathies (Alzheimer´s disease, AD; chronic posttraumatic encephalopathy, CPE), according to the tau-protein isoform in the “microtubule-binding” domain, its characteristics are coded by the specific mutations (variants) in the *MAPT* gene (locus 17q21.3).

### TDP-43 proteinopathies

TDP-43 proteinopathies are a group of diseases characterized by the presence of inclusions containing pathologically conformed TDP-43 protein (transactive-response DNA binding protein 43 kDa). Four additional subtypes are distinguished within TDP-43 proteinopathies based on the morphology and distribution of inclusions (type A–type D). Clinical manifestation is very heterogeneous within the FTLD-amyotrophic lateral sclerosis (ALS) spectrum. TDP-43 proteinopathies are most often associated with mutations in the genes *GRN* (progranulin), *TARDBP* (transactive response DNA-binding protein) and *C9orf72* (chromosome 9 open reading frame 72); more rarely in the genes *DCTN1* (dynactin) and *VCP* (valosine containing protein) (De Boer et al. 2021).

### FUS (fused-in-sarcoma) proteinopathies

FUS proteinopathies is a designation for a rare group of diseases with inclusions positive for the FUS protein. These are neuronal cytoplasmic and intranuclear inclusions, and in addition to neurons they can also be found in glial cells in the white matter. Within the FUS-proteinopathies, Basophilic Inclusion Body Disease (BIBD) and Neuronal Intermediate Filament Inclusion Disease (NIFID) are distinguished according to the characteristics of the inclusions. The clinical manifestation is also heterogeneous and includes clinical entities from the FTLD-ALS spectrum. Variants in *FUS* gene are responsible for this group of proteinopathies (Assoni et al. 2023).

## The importance of early differential diagnosis

The reason why the early recognition of the biological character of “future” neurodegenerative disease is so important is the hypothesis that the “disease-modifying therapies” are effective only in the prodromal/ presymptomatic period of the disease development (Kalia et al. 2024). In the total pool of patients suffering from neurodegenerative disease, those who suffer from TDP-43 proteinopathy and FUS and ubiquitinopathy form only a marginal part. The vast majority of patients suffer from either α-synucleinopathy or tauopathy. Therefore, the differential diagnosis is in almost all cases done between α-synuclein disease and tau-protein disease (see Table 2).

**Table 2:** Cellular processes and genes involved in neurodegeneration

Protein aggregation	Protein and membrane trafficking	Stabilisation of neurite structure	Prion-like transmission	Ubiquitin- proteasome system balance	Mitochondrial function and mitophagy	Lysosome autophagy	Synaptic function	Dopamine neurotran- smission
*SNCA*	*VPS35*	*LRRK2*	*SNCA*	*PRKN*	*PRKN*	*LRRK2*	*SNCA*	*SNCA*
*MAPT*	*DNAJC13*	*MAPT*		*FBXO7*	*PINK-1*	*VPS35*	*LRRK2*	*LRRK2*
	*LRRK2*			*SCA3*	*DJ-1*	*DNAJC13*	*SYNJ1*	*SYNJ1*
	*GAK*				*CHCHD2*	*ATP13A2*	*ATP13A2*	*ATP13A2*
	*RAB7L1*				*SREBF1*	*GBA*	*GCH1*	*GCH1*
	*RAB39B*					*SCARB2*		

## Genetic risk factors

The review research was completed according the SQAC methodology and PRISMA guidelines (Kmet et al. 2004; Tricco et al. 2018; Page et al. 2021). It seems that some authors refer to Parkinson’s disease, in its pathophysiological complexity, as a predominantly genetic disease. Beside the best known (and in fact very rare) Mendelian causes, there is a broad field of susceptibility variants and the synergic presence of polygenic pathophysiological factors (Cherian et al. 2023; Cocoș and Popescu 2024, Lim et al. 2024; Tenchov et al. 2025). These genetic risk factors can be stratified according to their dominance in the pathophysiological cascade and metabolic or structural processes which can lead to the cellular disturbance. The involved cellular processes embrace protein aggregation, membrane trafficking, stabilisation of the neurite structure, prion-like transmission, ubiquitin–proteasome system balance, mitophagy, lysosome autophagy, synaptic functions, and dopamine transmission (Kalia and Lang 2015, Ch). The most relevant genes/variants related to degenerative Parkinsonism, causative and risk/associated can be seen in Table 1. and they are also described below:

### Causative genes

#### *SNCA *(α-synuclein 4q21–23; *PARK1)*

The first locus encoding the misfolding of α-synuclein and manifestation of parkinsonian phenotype was reported in 1997: point mutation p.A53T (c.209G > A) of the *SNCA* gene (Polymeropoulos et al. 1997). There are now more mutations known; more frequent whole gene duplications or triplications and 22 (currently known) point mutations. The phenotype is usually quite similar to idiopathic Parkinson´s disease, only the onset is earlier: 4.–5. decade of life. The disease is characterised by the rapid progression and frequent presence of dementia, and patients regularly respond well to L-DOPA treatment. In both duplication and triplication cases the manifestation is atypical, accompanied by myoclonus and generalised dysautonomia. The spread of α-synuclein pathology is believed to be similar to prion diseases, and its extent and degree are reflected in the Braak´s staging of α-synucleinopathy (Breydo et al. 2012; Oueslati et al. 2014; Yi et al. 2025). In the general population, *SNCA* pathogenic variants are almost negligible in frequency (< 0.01%) and in cohorts with familial disease this frequency is up to 2% (Flores–Ocampo et al. 2025).

#### *MAPT* (tau)

*MAPT* gene encodes the microtubule-associated protein tau (MAPT) whose transcript undergoes complex, regulated alternative splicing, giving rise to several mRNA species. MAPT transcripts are differentially expressed in the nervous system, depending on stage of neuronal maturation and neuron type. This is reflected in the clinical phenotype: *MAPT* gene mutations are associated with several neurodegenerative disorders called “tauopathies”, such as Alzheimer's disease, Pick's disease, or frontotemporal dementia; cortico-basal degeneration and progressive supranuclear palsy are newly counted into one category (Stamelou et al. 2021; Goedert et al. 2023; Buchholz and Zempel 2024; Pedicone et al. 2024). Familial AD prevalence increases with age up to 20% of all cases among individuals over 65; hereditary frontotemporal dementia with Parkinsonism linked to chromosome 17 (FTDP-17) accounts for 5% of familial frontotemporal dementia cases, although its exact prevalence is unknown due to its rarity (Blass et al. 2025).

#### VPS35 (PARK17)

This gene belongs to a group of vacuolar protein sorting (VPS) genes. The encoded protein is a component of a large multimeric complex (retromer complex) involved in retrograde transport of proteins from endosomes to the trans-Golgi network. Mutations in the *VPS35* gene are associated with the manifestation of autosomal—dominant hereditary phenotype quite similar to idiopathic Parkinson´s disease; due to clusters in Austria and Switzerland the disease is counted among endemic Parkinsonism (Qui et al. 2024; Rowlands and Moore 2022; Struhal et al. 2014). Particularly, a single missense mutation in *VPS35*, Asp620Asn (D620N), segregates with late-onset disease in an autosomal dominant manner. The D620N mutation is the only mutation in *VPS35* with proven pathogenicity, however, several putative pathogenic mutations have been identified (P316S, R524W). Prevalence of *VPS35* variants ranged between 1 and 1.3% in autosomal-dominant PD cohorts and 0.3% in sporadic cases (Williams et al. 2022).

#### DNAJC13 (REM-8, PARK21)

This gene encodes a member of the Dnaj protein family whose members act as co-chaperones of a partner heat-shock protein by binding to the latter and stimulating ATP hydrolysis. The encoded protein associates with the heat-shock protein Hsc70 and plays a role in clathrin-mediated endocytosis. *DNAJC13* is a causative gene for some autosomal-dominant familial form of PD. The clinico-pathological phenotype is late-onset, dopa-responsive Parkinsonism with Lewy body pathology and neuronal cell loss in the substantia nigra, consistent with idiopathic PD (Yoshida et al. 2018; Follett et al. 2019). *DNAJC13* is not a frequent PD gene; in most populations its variants are found in ~ 0.5–1% of individuals suffering from PD (Gialussi et al. 2021).

#### LRRK2 (PARK 8)

This gene is a member of the leucine-rich repeat kinase family and encodes a protein with an ankyrin repeat region, a leucine-rich repeat (LRR) domain, a kinase domain, a DFG-like motif, a RAS domain, a GTPase domain, a MLK-like domain, and a WD40 domain. The protein is present largely in the cytoplasm but also associates with the mitochondrial outer membrane. Mutations in this gene are associated with autosomal-dominant familial L-DOPA-responsive Parkinson´s disease; *LRRK2* Parkinsonism with causal p.R1441G (c.4321C > G) mutation is endemic in Basque Country. One of the phenotypes is characterised by the absence of Lewy bodies in the pathological picture (Yakhine-Diop et al. 2022; Mensikova et al. 2023; Taymans et al. 2023; Sosero et al. 2023, Xiong et al. 2024). *LRRK2* has also been widely characterised for its major involvement in monogenic PD, the p.Gly2019Ser variant is the most common monogenic cause of PD, accounting for up to 4% of cases worldwide (Flores-Ocampo et al. 2025).

#### GAK

*GAK* (cyclin-G associated kinase) is an association partner of cyclin G and CDK (cyclin-dependent protein kinases); CDK governs the whole cell cycle. Cyclins are molecules that possess a consensus domain called the “cyclin box”. Variant rs1564282 (c.3283 + 1081G > A) in the *GAK* locus on chromosome 4p16 has been identified as significantly (OR = 1.28) associated with the manifestation of parkinsonian phenotype in Chinese population similar to idiopathic Parkinson´s disease (Li et al. 2021; Ng et al. 2024). In the past, there was a genome-wide significant association reported (P = 3.97 × 10^–8^) between a polymorphism, rs1564282, in the *GAK* gene and increased PD risk, with a meta-analysis odds ratio of 1.48 (Dumitriu et al. 2011).

#### RAB39B

Ras Analogue in Brain 39b (*RAB39B*) encodes a member of the RAB family of proteins. RAB proteins are small GTPases which are involved in vesicular trafficking; the genetic defect in *RAB39B* can cause endolysosomal dysfunction. Missense mutation p.T168K (c.503C > A) is associated with X-linked young-onset Parkinson´s disease with cognitive disability (Wilson et al. 2014; Koss et al. 2021; Yahya et al. 2023; Abusrair et al. 2023; Jacobson et al. 2024). *RAB39B* mutations are extremely rare, up to 20 individuals were reported with pathogenic variants; their overall prevalence is < 0.1% among PD patients (Mata et al. 2015).

#### RAB7L1 (PARK16)

Two variants in *RAB7L1* (Ras Analogue in Brain 7L1, rs947211 and 1572913), strongly associated with the manifestation of non-familial Parkinson´s diseases, lead to endolysosomal and Golgi apparatus sorting defects and deficiency of the *VPS35* component of the retromer complex. Expression of wild-type *VPS35*, but not a familial PD-associated mutant form, rescued these defects. However, *RAB7L1* is considered to induce strong susceptibility for manifestation of the disease (He et al. 2017; Liu et al. 2020). *RAB7L1* is not a causal PD gene, but common non-coding polymorphisms in its locus are consistently weakly protective (Cai et al. 2018).

#### RAB32

A novel monogenic risk factor which has been reported quite recently; the RAB32 p.Ser71Arg is a founder variant, which leads to the overactivity of LRRK2. Its mode of inheritance is autosomal-dominant with (supposedly) reduced penetrance. The phenotype is similar to sporadic dopa-responsive Parkinson´s disease with adult onset; the disease frequently manifests with severe non-motor symptoms (dysautonomia and hyposmia). Pathology is unknown; the seeding assay confirmed presence of misfolded α-synuclein in cerebrospinal fluid; the prevalence is still unknown (Gustavsson et al. 2024; Gustavsson 2025; Kleinz et al. 2025).

#### PRKN (PARK 2)

The encoded protein, parkin, is a component of a multiprotein E3 ubiquitin ligase complex that mediates the targeting of substrate proteins for proteasomal degradation, although the precise function of this gene is not fully understood. Alternative splicing of *PRKN* can result in multiple transcript variants encoding its different splicing; other splice variants of *PRKN* were described but still are lacking transcript support. The mutations in this gene are strongly associated with the familial, autosomal recessive, young-onset Parkinson´s disease (Choong et al. 2023, Funayama et al. 2023, Clausen et. al. 2024, Hattori et al. 2024). *PRKN* is probably the most common autosomal recessive juvenile PD gene, accounting for almost 28% of cases of European ancestry (Flores-Ocampo et al. 2025).

##### FBXO7 (F-box protein 7, PARK 15)

The *PARK 15* gene encodes one member of an extensive protein family sharing an approximately 40-amino-acid motif called F-box. The F-box proteins constitute one of the four subunits of the ubiquitin protein ligase complex called SCFs (SKP1-cullin-F-box), which function in phosphorylation-dependent ubiquitination. The family of F-box proteins is usually classified (according to presence of different amino acids) in three classes: *Fbws* which contain WD-40 domains, Fbls with leucine-rich repeats, and Fbxs with protein–protein interaction modules. The mutations in this gene are strongly associated with the unique phenotype manifesting with the autosomal-recessive familial disease merging the atypical parkinsonian syndrome with pyramidal signs, sometimes nicknamed “Parkinsonism—pyramids” (Nelson et al. 2013; Deng et al. 2013; Randle et al. 2017; Zhou et al. 2018; Mensikova et al. 2019; Navarro et al.^ 2024^). To date, only eight studies have identified cases carrying pathological *FBXO7* variants (Alizadeh et al. 2023).

###### *ATXN3/*SCA3

Spinocerebellar ataxia type 3 (SCA3), formerly described at Cape Verde Islands as Machado-Joseph disease (MJD, according to the surname of affected family) is characterized by progressive cerebellar ataxia and extrapyramidal symptoms like dystonia, rigidity and hypertonus of the lower limbs. Also, muscular atrophy, tendon hypo- or areflexia, and progressive ophthalmoplegia; sometimes fasciculations (typical for MND) might be present. SCA3 affects approximately 1:50,000–100,000 individuals and symptoms typically begin in adulthood between the third to fifth decades of life; the range of neurological symptoms widens with the progression of disease (Schöls et al. 2000; Durcan et al. 2013; Park et al. 2015a, b; Peng et al. 2019; Déglon 2024).

Parkinsonism has been described also as a rare clinical manifestation in the carriers of other SCA mutations: SCA2, SCA6, SCA8, SCA13, SCA15 and SCA caused by the *COA7* variant (Park et al. 2015a, b; Singh and Wu 2020; Ouchi et al. 2023; Baradd and Sankhla 2024).

#### PINK-1 (PARK 6)

This *PINK-1* (*PTEN-induced kinase 1*) gene encodes a serine/threonine protein kinase that monitors mitochondrial integrity by sensing disability status. PINK-1 cooperates with parkin to facilitate autophagic clearance of damaged mitochondria (process known as mitophagy). The PINK-1—parkin axis acts as the core machinery for mitophagy in neurons; any subcellular incompetence in this axis may lead to nigral dysfunction, which manifests with familial, autosomal recessive early-onset Parkinson´s disease (Dodson et al. 2007; Hauser et al. 2013; Merwe 2015; Tanaka 2020). 0.072 to 0.25% of patients with early-onset PD carry pathogenic variants in this gene (Flores-Ocampo et al. 2025).

#### DJ-1 (PARK7)

This gene, which contains eight exons, is located at the chromosome band 1p36.23 and encodes a protein composed of 189 amino acids, with seven β-strands and nine α-helices. It belongs to the peptidase C56 family; biochemical and structural evidence has shown that DJ-1 functions as a homodimer. It serves as a positive regulator of androgen receptor-dependent transcription. Its function is also to work as a redox-sensitive chaperone and sensor for oxidative stress. It might also protect neurons against oxidative stress resulting in the reduction in the rate of cell death. Mutations in *DJ-1* gene can cause the manifestation of familial autosomal recessive young-onset Parkinson´s disease (Sun and Zheng 2023; Lind-Holm Mogensen et al. 2023; Skou et al. 2024). At least 20 variants in *PARK7* are relatively uncommon being present in 1 to 2% of young-onset Parkinson´s disease (YOPD) cases (Flores-Ocampo et al. 2025).

#### CHCHD2; CHCH210 (PARK22)

The mutations in the *coiled-coil-helix-coiled-coil-helix domain containing 2 or 10* (*CHCHD2; CHCHD10*) have been found to be linked to Parkinson´s disease, amyotrophic lateral sclerosis, and frontotemporal dementia. Both genes belong to eukaryotic CX9C proteins characterized by four cysteine residues spaced ten amino acids apart from one another. The genes have important function to mitochondrial well-being, since they are negative regulators of mitochondria-mediated apoptosis: in response to apoptotic signals, mitochondrial levels of their proteins decrease, allowing BCL2-associated X protein to oligomerize and activate the caspase cascade. The mutations in *CHCHD2* can cause the manifestation of familial, autosomal dominant Parkinson´s disease, sometimes with young-onset (Imai et al. 2019; Ikeda et al. 2022; Shammas et al. 2023). Familial PD due to *CHCHD2* is very rare, affecting roughly up to 1% of familial cases; in sporadic disease, rare *CHCHD2* variants are found in less than 1% (Jiang et al. 2022).

#### SREBF1

*Sterol regulatory element binding transcription factor 1* (*SREBF1*), has a conserved function in promoting mitochondrial translocation of Parkin and subsequent mitophagy, *Fbox and WD40 domain protein 7* (*FBXW7*), and the next six components of the lipogenesis pathway. The identification of *SREBF1-*related dysfunctional mitophagy as a risk factor for the manifestation of “sporadic “PD suggests a common mechanistic link between autosomal recessive and sporadic disease. As an important molecular regulator for cellular lipogenesis, the mutation in this gene can cause the manifestation of “sporadic Parkinson´s disease “ (usually with familial aggregation) due to impact of dysfunctional mitophagy on its pathogenesis (Do et al. 2011; Yuan et al. 2018; Lou et al. 2019; Tejera–Parrado et al. 2019). Its variant rs11868035 reached genome-wide significance (p ≈ 5.6 × 10⁻⁸; OR ≈ 0.85).

#### ATP13A2 (PARK 9)

The mutations in *ATP13A2* are associated with Kufor-Rakeb syndrome, which is an extremely rare, familial, young-onset atypical Parkinsonism with autosomal-recessive inheritance. This hereditary disease has been first described in a Jordan family after which it has been named. *ATP13A* encodes a member of the ATPases subfamily; this protein transports inorganic cations and other substrates. For this gene, multiple transcript variants encoding different isoforms were described. Kufor-Rakeb syndrome manifests usually with rigidity, bradykinesia, chin tremor, ataxia, supranuclear vertical gaze palsy, dystonia, and cognitive deterioration, in some cases, it might resemble progressive supranuclear paralysis—Richardson´s syndrome. The important differential—diagnostic feature is neuroimaging: in patients with Kufor-Rakeb syndrome the iron depositions are symmetrically present in the basal ganglia on brain magnetic resonance imaging. This fact is the reason why Kufor-Rakeb syndrome is classified among the heterogenous group of “neurodegeneration with brain iron accumulation disorder” or “pallidal degenerations”; fewer than 150 affected individuals have been reported worldwide, so the estimated prevalence is < 1/1,000,000 (Yang et al. 2014; Park et al. 2015a, b; Jellinger 2022; Sikora 2024; Croucher and Fleming 2024).

#### GBA

According to the recent data from large cohorts worldwide, up to 15% of patients carry any of the associated mutations in the *GBA* gene. Therefore, the mutations in *GBA* are the most prevalent and most important genetic risk factor for manifestation of “sporadic” Parkinson´s disease. The *GBA* gene encodes a specific protein, so called lysosomal enzyme glucocerebrosidase (GCase), which serves as a stabiliser of glycosphingolipid homeostasis. *GBA*-associated Parkinson´s disease presents similarly to “idiopathic” or “sporadic” Parkinson´s disease, except the younger age of patients (but it is not YOPD!), frequent and more severe cognitive impairment and quick progression; all these attributes suggest the classification among atypical forms of Parkinsonism (Koros et al. 2024; Mazzetti et al. 2024; Skrahin et al. 2024). Different variants of *GBA1* displayed diverse frequencies in various populations, ranging between 1 and 5% in the PD cohorts (Funayama et al. 2023).

#### SCARB2 (LIMP-2)

Recent WGS studies provided strong evidence that mutations in *SCARB2* are associated with the manifestation of “sporadic” Parkinson´s disease, yet the precise mechanism has not been fully elucidated yet. The protein which is encoded by the *SCARB2* gene acts mainly in the process of glucocerebrosidase transport to lysosomes (Alecu et al. 2019; Bellomo et al. 2019; Kiechle et al. 2020). Rare coding variants are extremely uncommon with frequency < 0.1% (Huh et al. 2023).

##### SYNJ1 (synaptojanin1, PARK 20)

*SYNJ1* was discovered 30 year ago as a protein of 145 kDa molecular weight that interacts with the growth-factor receptor-bound protein 2 (GrB2), a phosphoprotein involved principally in the processes of synaptic vesicle endocytosis and recyclin; soon the close association of its missense mutations with the autosomal-recessive young-onset atypical Parkinsonism has been revealed, too. SYNJ1 is a lipid phosphatase which is richly expressed in the brain tissue. It has a crucial role in regulating cell homeostasis and in the functions of early endosomal trafficking. SYNJ1 regulation of molecular processes linked to dopaminergic transmission have not been fully elucidated yet. Biallelic nonsense mutations of *SYNJ1* are also associated (besides Parkinsonism) with Alzheimer´s disease and with severe neurodegenerative tauopathy manifesting with intractable seizures; so the phenotype of *SYNJ1* disease is significantly variable. The prevalence is extremely low, less than 0.1% of PD cases (Drouet et al. 2014; Dyment et al. 2015; Fasano et al. 2018; Ando et al. 2020; Choudry et al. 2021).

#### GCH1

The role of guanosine triphosphate (GTP) cyclohydrolase 1, encoded by *GCH1*, in the very first phase of tetrahydrobiopterin (BH_4_) cellular biosynthesis was revealed and described in 1996. Tetrahydropterin (BH_4_) is a crucial cofactor for dopamine synthesis in the cells of the substantia nigra pars compacta (SNpc), whose projections connect brainstem structures with the striatum. Mutations in the *GCH1* gene are causal for autosomal—dominant DOPA-responsive dystonia (DRD, DYT5) and very rare familial hyperalaninemia. Frequent co-occurrence of DYT5 and young-onset atypical Parkinsonism has been repeatedly observed in families with confirmed *GCH1* mutations; pathogenic *GCH1* mutations were the second most prevalent in big cohort studies of patients suffering from “sporadic” Parkinson´s disease, contributing to familial YOPD cases by 0.75–1.9% (Nagatsu et al. 1996; Mencacci et al. 2014; Yoshino et al. 2018; Saranza et al. 2021; Gupta et al. 2023; Makarious et al. 2023; Nagatsu 2024).

#### DCTN1

First described by Perry in Canada in 1975, the Perry disease is an autosomal-dominant hereditary neurodegeneration, which manifests in adulthood with Parkinsonism present together with psychiatric features (apathy and depression), central hypoventilation and sleep disturbance (Perry et al. 1975). The disease is caused by the exon mutations of dynactin 1 (*DCTN1*) gene; to date, 8 variants in 22 pedigrees have been reported. From the pathological point of view, it is a TDP-43 proteinopathy with dominant neuronal loss in the substantia nigra and locus coeruleus (Tsuboi et al. 2021).

### Risk/associated genes

In addition to the genes described above, tens to hundreds of other genes are currently thought to be involved in the pathogenesis of Parkinsonism. When selecting the phenotypes *Parkinsonism (****HP:0001300****), Parkinsonism with favorable response to dopaminergic medication (****HP:0002548****), juvenile-onset Parkinson´s disease (****MONDO:0000828****)*, ***progressive supranuclear palsy, multiple system atrophy and corticobasal degeneration*** then 217 genes would be associated with these phenotypes. This long list of genes contains merely risk genes identified by allelic association studies. However, it includes some genes that have been described in the previous paragraphs among Mendelian-acting genes (such as *SNCA* and *PINK1*); the reason is that some variants in the gene may act in a Mendelian fashion and others may increase the risk incrementally: *ABCA7, ADAR, ADH1C, AFG3L2, ALS2, ANG, ANXA11, AOPEP, AP5Z1, APOE, APP, ARVCF, ATN1, ATP13A2, ATP1A3, ATP6AP2, ATP7B, ATXN2, ATXN3, ATXN8OS, BMP2, C19orf12, C9orf72, CAT, CCNF, CFAP410, CHCHD10, CHCHD2, CHMP2B, CLN3, CLN5, CLN6, CLN8, COASY, COMT, COQ2, CP, CSF1R, CTSD, CYLD, CYP27A1, DAO, DCTN1, DNAJC12, DNAJC13, DNAJC5, DNAJC6, ****DUSP10,**** EIF2AK2*, ***EIF2AK3****, EIF4G1, ERBB4, FBXO7, FIG4, FMR1, FNIP1, FTL, FUS, GAA, GABRA1, GABRG2, GBA1, GCH1, GIGYF2, GLE, GLT8D1, GP1BB, GRN, GTPBP3, HIRA, HNRNPA1, HNRNPA1B1, HTRA2, IFNG, IMPDH2, JAG2, JAM2, JMJD1C, JPH3, KCNN2, KIF5A, LAMP2, LRRK2, LYST, MAPT, MATR3, MECP2, MFSD8, ****MOBP****, MPO, MRPS14, MT-ATP6, MT-CO1, MT-CO2, MT-CO3, MT-CYB, MT-ND1, MT-ND4, MT-ND5, MT-ND6, MT-TC, MT-TF, MT-TH, MT-TK, MT-TL1, MT-TQ, MT-TS1, MT-TS2, MT-TT, MT-TV, MT-TW, MTFMT, MYORG, NDUFAF1, NEFH, NEK1, NOS3, NOTCH3, NR4A2, NUP54, NUP62, OPTN, PANK2, PARK7, PCBD1, PCDH19, PDE10A, PDE8B, PDGFB, PDGFRB, PFN, PINK1, PLA2G6, PLAU, PODXL, POLG, PON1-3, PPARGC1A, PPP2R2B, PPT1, PRKAR1B, PRKN, PRKRA, PRNP, PRPH, PSAP, PSEN1, PSEN2, PTPA, PTRHD1, PTS, RAB39B, RREB1, ****RUNX2***, *SCARB2, SCN1A, SCN1B, SCN2A, SCN9A, SEC24C, SIGMAR1, SLC18A2, SLC20A2, SLC30A10, SLC39A14, SLC6A3, SNCA, SNCAIP, SNCB, SOD1, SORL1, SPG11, SPTLC1, SQSTM1, STUB1, ****STX6****, SYNJ1, TAF1, TAF15, TARDBP, TBK1, TBP, TBX1, TCAP, TH, TIA1, TK2, TMEM106B, TMEM240, TNNI3, TNR, TOMM40, TPP1, TREM2, ****TRIM11,**** TRPM7, TSC1, TSC2, TSPOAP1, TUBA4A, TWNK, UBQLN2, UBTF, UCHL1, UFD1, UQCRC1, VAC14, VAPB, VCP, VPS13A, VPS13C, VPS35, WARS2, WDR45, XPR1 ABCA7, ZFYVE26* according to Varsome Clinical 13.1.2 (Kopanos et al. 2018; Debnath et al. 2022).

## Environmental risk factors

There are numerous substances which have been accused of being an environmental risk for the development of neurodegenerative proteinopathy and Parkinsonism. These include mainly the agents used in agriculture and textile industry. The most important and most frequently studied are the pesticides and trichlorethylene.

### Pesticides

The most commonly mentioned group of substances are the pesticides*. The term “pesticides”, apparently derived from “pest”, is an umbrella term for three classes of toxic substances, that in agriculture regulate unwanted organisms, i.e. insects, harmful plants, and fungi: insecticides, herbicides, and fungicides. The relationship between occupational exposure to pesticides and Parkinsonism became a popular topic at the start of the eighties, when the small epidemic of post—MPTP Parkinsonism was documented. MPTP (1-methyl-4-phenyl-1,2,3,6-tetrahydropyridine) has the similar structure as paraquat, one of the most potent herbicides, and they are both substituted pyridines. Paraquat exposure was probably the most frequently mentioned environmental cause of Parkinsonism, followed by the exposure to dithiocarbamates, rotenone, glyphosate, pyrethroids, organochlorine, organophosphates (terbufos) and trifluralin (Ball et al. 2019; Yuan et al. 2021; Tong et al. 2022; Tsalenchuk et al. 2023; Brolin et al. 2024).

### Trichloroethylene

Trichlorethylene used to be (and still is) a solvent used in numerous industrial processes, namely in the chemical and textile industry. It is an easily water-soluble substance. It is extremely ubiquitous and it can be found everywhere in both the environment and human body: in the soil, in drinking water, in the blood, urine, CSF and even maternal milk. The main metabolic pathway of trichlorethylene is cytochrome CYP450 oxidation, and the metabolites have mutagenic properties; the most frequently mentioned disease linked to trichlorethylene is cancer. The first reports about trichlorethylene neurotoxicity came from the second half of the last century, and were dedicated mainly to “neurodegeneration”. Parkinson´s disease has been linked to trichlorethylene exposure quite recently, because a statistical association exists between PD and trichloroethylene exposure at Camp Lejeune, a military base in North Carolina, United States (De Miranda 2020; Dorsey et al. 2023; Goldman et al. 2023). Trichlorethylene can cross the blood—brain barrier and accumulates in the nervous tissue and CSF. Still, the toxicological, genetic and epidemiological prospective studies in large exposed cohorts are still lacking. Nevertheless, the evidence from animal studies showed that the exposure to trichlorethylene leads to dopaminergic neuron loss and development of α-synucleinopathy in surviving ones, resulting in motor and gait impairments (Adamson et al. 2023). The probable mechanism of trichloethylene neurotoxicity is the reduction of activity of mitochondrial complex I enzyme, the mechanism similar to that described in Caribbean endemic Parkinsonism (Sauerbeck et al. 2012).

## Environmental risk factors in endemic Parkinsonism

Besides the globally ubiquitous substances, which are supposedly neurotoxic and exposure to them can cause the manifestation of Parkinsonism, there are more geographically (regionally) specific substances, which cause (or until quite recently caused) the manifestation of endemically present Parkinsonism. Among ten types of endemic Parkinsonism, there are three of them in which an environmental cause is believed to be presumable: Western Pacific Parkinsonism, Caribbean Parkinsonism, and North France cluster of atypical Parkinsonism (Mensikova et al. 2023).

### Western Pacific foci of endemic Parkinsonism

The presence of endemic disease amyotrophic lateral sclerosis—Parkinsonism—dementia (ALS/PDC) has been documented in three spots in the Western Pacific region: the island of Guam, Kii peninsula of the Honshu Island in Japan and in the villages of lowlands of west Papua–Irian Jaya (Spencer et al. 1991). The first reports from Guam came from Navy pathologists and later from Navy neurologists in the years 1945–1953 (Cox et al. 2002). In the sixties came first reports from Kii in Japan (Spencer 2020). The disease in Irian Jaya has been initially studied by Gajdusek between 1962 and 1967 (Gajdusek et al. 1982). The phenotype at all three Pacific spots was similar: the combination of symptoms typical for ALS, Parkinsonism with dementia or dementia alone. In the majority of cases, the symptoms of one of the abovementioned diseases prevailed (Marler et al. 2005; Okumiya et al. 2014). The pathology of Guam complex and Kii disease was similar: the “mixed pathology” with features of α-synucleinopathy, tau pathology (neurofibrillary tangles, NFT) and pathology typical for ALS, including their hallmarks: Hirano bodies and Lewy bodies (Steele et al. 2008). The pathology of Irian Jaya ALS/PDS was never available (Spencer et al. 1987). The pathophysiology of all three Pacific types of endemic Parkinsonism has been studied in detail for years. The hereditary origin was never confirmed. The environmental hypothesis soon prevailed: the consumption of fadang, seeds from the cycad tree *Cycas micronesica* has been accused (Spencer et al. 2005; Steele et al. 2008). *Cycas micronesica* contains two potent neurotoxins: methylazoxymethanol glucoside (cycasin) and the beta-methylamino-L-alanine, shortened as *MAM* and *BMAA*, respectively*.* The hypothesis of biomagnification of toxin levels at the island of Guam in the tissue of the Mariana fruit bat, *Pteropus mariannus*, have not been finally confirmed (Mensikova et al. 2023).

### Caribbean foci of endemic Parkinsonism

The presence of endemic disease resembling either Parkinson´s disease or progressive supranuclear palsy has been documented in two spots in the Caribbean: the island of Guadeloupe (1999), and later the island of Martinique (2018). The first reports from Guadeloupe came from the young neurologist Dominique Caparros–Lefebvre, working at the University department of neurology in Pointe-de-Pitre (Caparros 1999, 2002, Steele et al. 2002). After her return to France, the on-site research was run by Annie Lannuzel (Lannuzel 2007). The initial cohort at Guadeloupe was re-examined in 2006: only one fourth of probands suffered from Parkinson´s disease, and three fourths suffered from other Parkinsonism: either Guadeloupean PSP-like syndrome, Guadeloupean Parkinsonism/dementia complex or other form of atypical Parkinsonism (Caparros 2006).

Almost twenty years later, the disease has been identified at the island of Martinique where the phenotype ratio was almost similar to those at Guadeloupe. The authors of the final report suggested to use the term “Caribbean Parkinsonism” for this disease (Lannuzel 2018). The pathology of Caribbean Parkinsonism found at both islands was practically similar: the “mixed pathology” with features of dominant tau pathology with concomitant α-synucleinopathy; in the PSP-like cases with prominent supranuclear gaze palsy, the pathology was identified as a pure tauopathy (Mensikova et al. 2023). Hereditary origin was excluded (Camuzat et al. 2008). Soon after (in fact already in the first report) the environmental hypothesis was postulated: the consumption of soursop, the fruit of the tropic plant Annona muricata, consumed in the form of juice or herbal teas. This part of the plant contains the acetogenin called annonacin, one of the most potent inhibitors of the mitochondrial complex I (Lannuzel 2008; Spencer 2017; Kedari et al. 2014).

### North France cluster of atypical Parkinsonism

Caparros–Lefebvre and Golbe reported in 2015 the existence of a cluster of progressive supranuclear palsy (PSP) in Northern France (Hauts-de-France province). This cluster lay in the districts of the towns Wattrelos and Leers (total population of both towns with surrounding villages where the PSP cases lived was around 60,000). There were altogether 92 PSP cases identified, from which 62 lived directly in both towns. The patients were examined in detail, including molecular genetic study. 43% of probands suffered from PSP-R, and 42% suffered from PSP-P, 15 probands manifested symptoms of other PSP phenotypes; the mean disease duration was 5.74 years. None of the probands reported the occurrence of PSP or other phenotype of Parkinsonism in the family. There were 13 probands autopsied, and on autopsy typical tau brain pathology was found, including severe neuronal loss predominantly in the substantia nigra and locus coeruleus, and diffuse tau protein deposits. Globoid neurofibrillary tangles and tufted astrocytes were frequent in the substantia nigra, subthalamic nucleus, locus coeruleus, midbrain tegmentum, cerebellum, and pallidum. The molecular-genetic study was done in twelve autopsied cases. Of the 24 chromosomes 17 s in these cases, 23 (96%) bore the H1 haplotype. The frequency of allele A of SNP rs242557 occurred in 66.7%. All of the frequencies were similar to those previously described in PSPwith an overrepresentation of the H1 haplotype and H1c subhaplotype. An environmental origin of this PSP cluster has been suggested (Caparros–Lefebvre et al. 2015). Suspicion was directed at the site just south of the center of Wattrelos, where large heaps of spent ore (slag) from the now-defunct metals extraction industry are located. Other locations in the immediate area may have been contaminated by the numerous textile-dyeing and leather-tanning plants that used metals in their processes. The most suspicious neurotoxic agents considered were arsenic and chromium, independently or in synergy. This hypothesis was supported by the findings of a study conducted with cell models (neuroblastoma cell lines), in which the exposure to chromium and nickel induced cell death with typical tau changes (Alquezar et al. 2020).

## Discussion

Of course, it is a bit of tricky task to review genetic and environmental risk factors in the situation where the debate about the nosological nature of the term “Parkinson´s disease” eagerly continues, and nowadays, it is not only the debate about the “biological staging system”, i.e. the SynNeurGen v.s. NSS-ISS (Hoglinger et al. 2024; Simuni et al. 2024). It is also the debate about real content of the term itself. Is PD really a disease, or just a syndrome, as has been proposed by Donald Calne and Bill Weiner (Calne 2001; Weiner 2008) twenty years ago?

This nosological entity shares important characteristic properties with other nosological categories: the cases usually have similar pathology (Lewy pathology with Lewy bodies, formed by misfolded α-synuclein), the cases usually have almost similar clinical presentation, the cases usually respond to the same drug (L-DOPA), the cases usually have almost identical life expectancy. On the other side, there are numerous characteristics which differ from other nosological categories: numerous cases have a common phenotype and quite uncommon pathology, still they respond well to the same drug (L-DOPA). Numerous cases have common Lewy-body pathology and an uncommon phenotype, either oligosymptomatic (benign tremulous Parkinsonism, gait ignition failure) or differently symptomatic (dementia, generalised dysautonomia). So, it is very hard to define whether is it a disease or just a syndrome. The validated clinical diagnostic criteria for Parkinson´s disease still exist and are still in routine use (Gibb 1988; Postuma 2015), the important clinical trials completed in last 30 years have used these criteria for recruitment.

The knowledge gap in the abovedescribed topics is, despite the indisputable and permanent new scientific acquisitons, still huge. There are many fields, items and topics which deserve further research and incorporation into the whole concept of the neurodegenerative pathophysiology. However, one thing remains clear: loss of neurons and its clinical rebound are only the very end of the whole process. Its most important steps are neuroinflammation and subsequent neurodegeneration with pathological proteins deposit; both are probably caused by the etiological interplay between genetic and environmental factors.

### Neuroinflammation

The role of neuroinflammation in the pathophysiology of neurodegeneration has become a central focus of research (including for ourselves) in the recent years, with permanently increasing evidence that neuroinflammation itself is probably the key factor in the triggering and progression of neurodegeneration. Nevertheless, some issues remain still unresolved: whether neuroinflammation is primarily protective or harmful, and in what step of the process is the balance between these actions finally disrupted. The term “neuroinflammation” itself refers to a broad scale of immmune responses which are triggered when any trouble is detected within the central nervous system (CNS). This covers rather typical inflammatory responses, with the activation of complement, release of cytokines and chemokines, and the activation of the local immune cells, including microglia and astrocytes. Circulating immune cells usually infiltrate the CNS and immune dysregulation arrives within the brain of individuals which eventually develop Parkinson´s disease. Significantly elevated levels of pro-inflammatory cytokines have been detected in the blood serum of these patients, suggesting that they are directly associated with the onset of the neuroinflammation process (Adamu et al. 2024; Cook et al. 2025; Malpetti et al. 2025; Müller and DiBenedetto 2025). On the other side, there are different opinions in which “neuroinflammation “ is denoted as an “abused “ term, the concept itself is marked as masking gliopathies; some authors even expressed their belief that the immune signaling in CNS circuits is not neuroinflammation etc. (Galea and Graeber 2023; Socodato and Relvas 2025). These controversies may be solved only with the new datasets from large prospective cohort studies, which should also finally elucidate the justification of brain-first versus body-first connectome concept (Horsager and Borghammer 2024).

### Neuropathology (including the role of pathological proteins)

The quite recent evidence of mutiple brain pathology presence in the majority of autopsied cases has brought another dimension into this heated debate (Forrest and Kovacs 2023, 2025). The current concept of neurodegenerative diseases classification has practically torn-off the classical and “appropriately ordered” nosological categories, leaving the pathological and clinical diagnostic algorithms useless. It would therefore be fair to state here that we are reviewing the genetical and environmental risk factors of neurodegenerative Parkinsonism rather than Parkinson´s disease, in this case “familial and sporadic α-synucleinopathy”. Although the general opinion prevails that the pathological proteins (α-synuclein, tau-protein, ubiquitin, TDP-43 protein, FUS-protein) are the products of altered neuronal or glial metabolism and interfere with the normal cellular function, some authors questioned this concept and hypothesised about the biological and protective role of these proteins, particularly α-synuclein (Espay and Lees 2024a, b; Espay et al. 2025).

### Interplay between genetic and environmental factors

It is probably clear that pure forms of “genetic” Parkinsonism exist, caused by the presence of a causal variant in the given gene; this type of the disease is represented by several forms of monogenic, mendelian disease (*SNCA1, PINK1, VPS35* etc.). It is probably similarly clear that pure forms of “environmental” Parkinsonism exist, caused by the acute or chronic poisoning by well-known toxic agents (MPTP, methanol, manganese, CO etc.). Between these poles stands a broad field of disease types which are caused by the interplay between genetic and environmental factors. There are many genes with many variants which increase the “susceptibility” to become ill with Parkinsonism. The majority of recently described variants in numerous genes simply predispose to Parkinsonism, and the way to the disease is through another vector, which is likely environmental. These group of genes include those described in subheading 3b., while the most notorious are probably *ATP13A2*, *C9orf72*, *DCTN1*, *GBA1*, *GIGYF2*, *GRN*, *HTRA2*, *PANK2*, *PLA2G6*, *PRNP*, *RA39B*, *SLC18A2*, *SREBF1*, *SCARB2*, *SYNJ1*, *TAF1* and many others. Interestingly, some mendelian, autosomal-dominant and highly penetrating variants are also mentioned in association with the robust environmental risk factors, such as paraquat, rotenone, trichlorethylene. For instance, *SNCA* has been associated with the exposure to paraquat and rotenone, *LRRK2* to rotenone and trichlorethylene, and *VPS35* to rotenone. This evidence would lead to the hypothesis that these variants may become fully penetrant only through interaction with toxins in the environment (Bogers et al. 2023). Therefore, with the hypothetic exception of acute poisoning, the manifestation of Parkinson´s disease (or neurodegenerative Parkinsonism, whichever) would be possible only with the co-existence of both types of risk factors—genetic and environmental (Jacobs et al. 2020; Perinan et al. 2022; Reynoso et al. 2024). Also, the results of recent clinical trials based on the genetic determination of Parkinsonism are another indirect evidence of that (Prasuhn and Brügemann 2021; Sjögren et al. 2021; Denaro et al. 2024). According to our knowledge, to date, there are several big cohort studies running or in preparation which will focus excellently on this topic; let´s hope there will be a more clear picture within the next few years.

## Conclusions

There are many factors which have significant impact on the development and clinical manifestation of neurodegenerative proteinopathies. While the evidence of important genetic and environmental risk factors is growing, the evidence of their mutual interplay is still scarce (Bogers et al. 2023). In the future, sophisticated genomic/exposome research in large cohorts would have the potential to reveal these relationships and describe their supposed reciprocal influence.
